# Processing and acquisition of temporality in L2 Mandarin Chinese: Effects of grammatical and lexical aspects

**DOI:** 10.3389/fpsyg.2022.964861

**Published:** 2022-09-15

**Authors:** Shaohua Fang, Yi Xu

**Affiliations:** ^1^Department of Linguistics, University of Pittsburgh, Pittsburgh, PA, United States; ^2^Department of East Asian Languages and Literatures, University of Pittsburgh, Pittsburgh, PA, United States

**Keywords:** processing, Mandarin Chinese, lexical aspect, grammatical aspect, second language acquisition

## Abstract

This study investigated the second language (L2) processing and acquisition of Chinese temporality, specifically the interaction of grammatical and lexical aspects. An experimental group of 31 English-speaking learners of Chinese and a control group of 29 native speakers of Mandarin Chinese completed an online sentence-picture matching task and an offline translation task. Results from these experiments demonstrated the prototype effect: In aspectual development, perfective aspect started with telic verbs and progressive aspect started with activity verbs, in accordance with the Aspect Hypothesis, both for online processing and offline comprehension. The prototype effect of the grammatical aspect was evident for activity verbs but less so for accomplishment verbs in the L2 group across tasks, and this was explained through language-specific properties and L2 learners’ instructional input. In addition, L2 proficiency and working memory capacity were found to modulate these processes.

## Introduction

Time is crucial in human experiences and temporality can be expressed through a variety of mechanisms in different languages including adverbs, semantic features, syntactic structures, and discourse-pragmatic markers. In acquiring tense-aspect at the level of morphosyntax, learners are expected to derive the temporal interpretation for a given aspectual morpheme and/or its association with aspectual information separately encoded in lexical items. In this realm, “tensed” languages such as English have traditionally received more attention than “tenseless” languages (e.g., Li et al., 2022), yet it would be especially informative to investigate “tenseless” languages such as Mandarin Chinese (henceforth Chinese) so that the effect of aspect can be teased apart from that of tense. It is also timely to investigate the universal existence of time in human languages through the lens of Chinese. As the recent two decades witnessed a growing field of Chinese as a second or foreign language both in teaching practice and in theoretical development, scholars have pointed out how Chinese as a Foreign Language (CFL) research approaches should be informed by studies of other languages and how findings in CFL inquiries can advance second language acquisition and teaching by providing new evidence and testing the generalizability of existing theories (Gao et al., 2014; Gong et al., 2020a). In Gong et al.’s (2020a) review of CFL studies published in the most impactful journals in mainland China in 2014–2018, the authors pointed out that the learning of grammatical features is a key area in CFL teaching and research and they referred to studies on grammatical aspect learning as examples. The current article responds to this call for cross-linguistic perspectives in CFL research (e.g., Gong et al., 2020b) by supplementing the existing language acquisition theories regarding time expressions in human languages’ event structures and by taking advantage of the unique typological features in Chinese.

Across different languages, grammatical aspects can be encoded through morphological markers and lexical aspects are realized lexically (Comrie, 1976; Smith, 1997). It has been posited that grammatical and lexical aspects are associated in some principled fashion, as formulated in the well-known Aspect Hypothesis (henceforth the AH; Shirai and Andersen, 1995; Bardovi-Harlig, 2000). This hypothesis has its origin in first language (L1) acquisition (Andersen and Shirai, 1996) and has been extensively tested across languages among children (e.g., Shirai, 1998; Stoll, 1998; Li and Shirai, 2000; Chen and Shirai, 2010). Although the acquisition of tense-aspect morphology has been the focus of inquiry in the field of second language acquisition (SLA) for decades, especially through the functional approach, existing research has devoted a disproportionate amount of attention to perfective aspect only (Bardovi-Harlig, 2012). Moreover, relative to L1 acquisition research, SLA studies so far have addressed to a lesser extent the influence of lexical aspect and its interaction with grammatical aspect (*cf.*, Ryu et al., 2015; Zeng et al., 2021). In SLA studies of Chinese, there is a sizable body of literature on grammatical aspect, especially for learners’ knowledge and usage of the perfective marker -*le* (e.g., Wen, 1995; Duff and Li, 2002; Xu, 2020), but only a few studies have focused on the interactive effects of grammatical and lexical aspects (e.g., Jin and Hendriks, 2005). General SLA studies addressing the AH also showed mixed results, arguably due to task effects, with existing research relying predominantly on offline production tasks (Bardovi-Harlig and Comajoan-Colomé, 2020, p. 1158).

With these research gaps in mind, the current study aimed to investigate the effects of grammatical and lexical aspects on the processing and acquisition of the temporal structures of events in sentences by English learners of Chinese through both online and offline measures. We also explored the influence of individual differences in language proficiency and working memory capacity on these processes.

## Aspect hypothesis in relation to the current study

Grammatical aspect, also known as viewpoint aspect, reflects how a speaker views a situation/event. Perfective aspect (e.g., *have done* in English) is used when a situation is conceived as being completed (e.g., *We have talked*) and imperfective aspect (e.g., −*ing*) is used when a situation is conceived as being ongoing (e.g., *We are talking*). Commonly seen Chinese grammatical aspect markers include -*le*, -*zhe*, -*guo*, and *zai*, which encode the perfective, durative, experiential, and progressive aspects, respectively. Lexical aspect, also known as situation aspect, refers to the inherent temporal properties of a situation. Typical categories of lexical aspects include states, activities (ACTs), achievements, and accomplishments (ACCs) (Vendler, 1967). Different classes of lexical aspects can be identified with reference to, among other things, telicity and punctuality (Comrie, 1976; Dowty, 1979; Smith, 1997). For instance, based on telicity, ACT verbs and ACC verbs can be distinguished: While both involve a duration ([−punctual]), the two are distinct in that atelic ACTs (e.g., *run, swim*) do not have a natural endpoint and telic ACCs (e.g., *draw a picture*, *run a mile*) have an endpoint.

The grammatical and lexical aspects under investigation are relevant to two generalizations made in the AH. Specifically, the AH states the following regarding perfective and progressive markings in associations with different lexical aspects (Andersen and Shirai, 1996, p. 533):

1. Learners first use past marking (e.g., English) or perfective marking (Chinese, Spanish, etc.) on achievement and accomplishment verbs, eventually extending its use to activity and stative verbs.
2. In languages that have a progressive aspect, progressive marking begins with activity verbs, then extends to accomplishment or achievement verbs.

Shirai (2004) explains that the AH has two components. One component concerns the prototypical association between grammatical and lexical aspects in that learners tend to associate the perfective aspect marker with telic verbs such as accomplishments and achievements and the progressive aspect marker with atelic verbs such as activities. The other component pertains to aspectual development in language acquisition, which predicts that beginning learners are more constrained by the prototypical association than proficient learners. In other words, as their proficiency increases, learners will spread the use of the perfective aspect marker from telic verbs to atelic verbs, as well as extend the use of the progressive aspect marker from activities to telic verbs. These tendencies were first observed in L1 acquisition and have then been applied to the SLA of different languages including English, Spanish, Russian, Japanese, etc. (Andersen and Shirai, 1996). There have been a few related accounts that explain these tendencies. Anderson (1993) proposed the Semantic Congruence Principle on which the prototypical combinations of certain aspects semantically operate. For example, progressive markers and activities are semantically congruent, so there may be a universal predisposition for humans to give a progressive marking to notions associated with the [+durative] [−telic] features (e.g., *John is running*). Perfective aspect markers and accomplishments are also semantically congruent, as they are relevant to telic and bounded events. Shirai (2002) explains that if the prototypical semantic features (e.g., [+durative], [−telic]) are involved, the relevant form (e.g., progressive form) is easily activated and thus frequently produced in learners’ speech. The non-prototypical forms do not easily reach the threshold of activation to be accessed and processed during language comprehension and are less likely to be produced by learners. Similarly, non-prototypical forms tend to be acquired later than the prototypes. According to Shirai (2010), the underlying motivations for this prototype effect may also be closely related to input frequency. For instance, if the progressive marking occurs more often in ACTs and ACCs and less frequently in achievement and state predicates (e.g.,?^1^ *John is knowing*), then the distributional bias may affect acquisitional tendencies (Andersen and Shirai, 1994).

Although some existing studies, several of which reviewed below, show support for the hypotheses, divergent findings have also appeared, likely due to experimental designs. Whereas the AH predicts that the prototypical association between grammatical and lexical aspects would become less restrictive as learners’ proficiency increases, manifesting in areas such as the extension of imperfective across ACTs and ACCs, Shirai (2004) posited that such a developmental prediction was borne out more often in close passages than in cross-sectional and longitudinal studies for narratives. Moreover, with only a few exceptions (e.g., Chan, 2012; Roberts and Liszka, 2013; Zeng et al., 2021), most studies rely heavily on offline measures for researching L2 acquisition of aspects. Relatively little is known about how learners deploy aspectual information online for temporality to be computed during processing.

## Aspect in language processing

For the inquiry into the processing of aspects among monolinguals, the focus has been on the role of grammatical aspect in constructing the structural representation of an event (e.g., Altmann and Kamide, 2007) and more recently on the interplay between grammatical aspect and lexical aspect during sentence comprehension (e.g., Yap et al., 2009; Becker et al., 2013). A staple approach to tap into language processing on this topic is the use of online tasks, often with two contrasting visuals so that participants can match sentences with the visual information that depicts the situation. For example, Altmann and Kamide (2007) compared eye-movements for sentences such as *the man will drink* … or *the man has drunk* … in a visual scene either depicting a full glass of beer or an empty wine glass. They found more looks toward the full glass of beer in the future tense condition and more looks towards the empty wine glass in the past tense condition, suggesting that English participants made use of tense information to predict upcoming materials.

Grammatical aspect has been shown to affect language comprehension and processing, and the effect may surface differently depending on tasks. For example, in Magliano and Schleich (2000), native speakers of English read stories in which a situation was described either as ongoing or completed, and then were asked to decide whether a target verb phrase occurred in the prior story sentence. Their results showed that imperfective aspect has an advantage to facilitate participants’ correct decisions over perfective aspect: When participants had just read stories in imperfective situations, they responded faster to the target verb phrase than in conditions where they had read perfective situation stories. The authors attributed this imperfective facilitation to the slower decay rate associated with imperfective situations compared to perfective, arguing that an imperfective situation remains at a higher state of activation over subsequent context to be maintained and processed in working memory. Madden and Zwaan (2003), using a sentence-picture verification task, found that native speakers of English chose either pictures depicting ongoing events (e.g., *The man was making a fire*) or completed events (e.g., *The man made a fire*) when reading imperfective sentences, while they were more likely to choose pictures depicting completed events when reading perfective sentences. Of theoretical interest is the perfective facilitation effect found in Madden and Zwaan (2003) in contrast with the imperfective facilitation effect found in Magliano and Schleich (2000). How might we account for these two different effects? Apart from a potential effect from task variation, lexical aspect could also have been a confound, given that it had not been properly controlled for in either study, making the results of both studies more difficult to interpret and compare.

To examine if aspectual asymmetries would be modulated by lexical aspect, Yap et al. (2009) manipulated both grammatical and lexical aspect in a study of native speakers of Cantonese. In a sentence-picture matching task, participants decided which of the two pictures matched what they read. Crucially, perfective sentences were processed more quickly and accurately with ACC verbs than with ACT verbs, and imperfective sentences were processed more quickly and accurately with ACT verbs than with ACC verbs, demonstrating perfective facilitation for ACC verbs and imperfective facilitation for ACT verbs. Yap et al. (2009) argued that these patterns resulted from the more prototypical and frequent types of association between grammatical and lexical aspects and that such an association is mainly driven by the semantic congruity: Similar features reinforce each other, and dissimilar features result in slower processing. That is, the inherent telicity feature in ACCs is matched by the boundedness feature in perfective markers, and the inherent durativity feature of ACTs is matched by the ongoingness feature of the imperfective marker. Yap et al. (2009) further suggested that the interaction between different grammatical aspect markers and different verbs lead to differences in “neural activations” (p. 593). Thus, compatibility or congruity in features enables faster cognitive processing.

## Aspect in second language acquisition

The L2 acquisition of tense-aspect has been an area of intensive investigation for decades based on both a generative approach (e.g., Montrul and Slabakova, 2003; Gabriele, 2009) and a functional approach (e.g., Bardovi-Harlig and Reynolds, 1995; McManus, 2013). Within the functional framework, testing the validity and generalizability of the AH has been a central issue (see Bardovi-Harlig and Comajoan-Colomé, 2020 for a comprehensive review). Despite much empirical evidence, the AH is far from being settled. One apparent reason is that the acquisition and development of tense-aspect is a complex task and process, constrained by multiple factors, including the prototype effect, L1 transfer, and L2 input (Shirai, 2004). The other reason might be that such studies used different methods and targeted different languages (e.g., Bardovi-Harlig and Reynolds, 1995; Collins, 2002; Comajoan, 2006; McManus, 2013; Ryu et al., 2015), leading to mixed results. For example, Sugaya and Shirai (2007) addressed whether acquisition of the imperfective marker *-te i-ru* in Japanese was influenced by the lexical aspect using the acceptability judgment experiment. Their result supported the AH, as learners of Japanese were more likely to use *-te i-ru* with ACTs than with ACCs. The association between an imperfective marker and progressive interpretation was also observed in learners of Korean (e.g., Lee, 2001; Lee and Kim, 2007; Ryu et al., 2015). However, another series of studies showed results that were inconsistent with the AH (e.g., Salaberry, 1999, 2000; Labeau, 2005; McManus, 2013). For example, McManus (2013) used a spoken narrative task and a sentence interpretation task to uncover the development of aspect by English and German learners of French. While their results showed that learners in general were biased towards the prototypical associations predicted by the AH, their advanced level learner group was shown to be affected by the prototypical association to a larger extent compared to the low proficiency learner group, a pattern contradictory to predictions of the AH. To explain the results, McManus argued that the effect of AH would not show up until learners had achieved stability in mapping the meanings of perfective/imperfective markers to their obligatory contexts and only advanced proficient learners in their study used these markers in their relative obligatory contexts consistently, in a way similar to their native speaker control group. In turn, the author suggested that their advanced learners’ greater exposure to L1 naturalistic input may be responsible: If the prototypical associations are largely a result of frequency distribution bias, as suggested by Shirai (2010, pp. 184–186) and Hendriks (1999), then the advanced group, who declared more study abroad experience than the low-proficiency group, would be more affected by the distributional bias. McManus (2013) also suggested the possibility of a U-shaped development, claiming that the low proficiency group might already be too proficient to show stronger prototypical associations than the advanced group (p. 319).

From the language acquisition point of view, it is especially important to validate the generalizability of the AH by examining typologically different languages. Similar to the above-mentioned studies that show inconsistent developmental patterns regarding the agreement to the AH, both L1 and L2 acquisition studies testing Asian languages have yielded a picture of mixed results regarding the degree of support for the AH, with some demonstrating consistent results (e.g., L1 Chinese: Chen and Shirai, 2010; L1 Korean: Ryu and Shirai, 2022; L2 Japanese: Shirai, 1995; Shirai and Kurono, 1998; L2 Korean: Lee, 2001; Lee and Kim, 2007; Ryu et al., 2015; L2 Chinese: Jin and Hendriks, 2005) and others being not entirely consistent with the hypothesis (e.g., L2 Japanese: Sugaya and Shirai, 2007; L2 Chinese: Liu, 2012; Tong and Shirai, 2016).

There are a few areas where the current L2 literature on the AH predictions is particularly lacking. First, most previous studies used production tasks, with only a few that included comprehension (e.g., judgment) tasks. Task effect may be a factor that contributes to the variation in results (Shirai, 2004; Bardovi-Harlig and Comajoan-Colomé, 2020). For example, Sugaya and Shirai (2007) examined the L2 acquisition of Japanese imperfective aspect using an acceptability judgment task and an oral picture description task. They found that only the results from the judgment task but not from the oral production task supported the AH. One possible reason could be that their L2 participants were not yet ready to express a consistent sensitivity to aspectual asymmetries in production tasks yet, since production skills are often more delayed than judgment or comprehension skills. Second, most existing L2 studies that probed learners’ comprehension used offline tasks only (e.g., Sugaya and Shirai, 2007; Liu, 2012) and online measures were rarely adopted. As learners may draw on different types of knowledge (online for implicit knowledge vs. offline for explicit knowledge) for a task depending on whether the task is timed or not (Loewen, 2009; Godfroid et al., 2015), the use of an online task will minimize learners’ reliance on metalinguistic reasoning or prescriptive rules memorized through explicit instruction and enable us to better tap into L2 learners’ mental representations and processing of temporality. Finally, Bardovi-Harlig and Comajoan-Colomé (2020) mentioned that individual differences in language proficiency on the L2 acquisition of aspect have been intensively investigated. However, to the best of our knowledge, the role of working memory capacity (WMC) as an additional factor in individual variation has never been explored on the L2 processing and acquisition of aspect, although it has been examined in the processing of English past tense (Rızaoğlu and Gürel, 2020). The influence of WMC on individual differences in L2 processing in general has been well-documented (Linck et al., 2014; Wen, 2015) and a thorough investigation of learners’ performances should consider both their language proficiency and WMC effects. The present study seeks to fill the above methodological gaps. More specifically, we will examine the L2 processing and acquisition of Chinese in relation to the AH because the “tenseless” nature of Chinese enables us to reveal interactions between the grammatical and lexical aspects unconflated by tense.

## Aspect in Mandarin Chinese

With the lack of linguistic mechanisms in tense, temporality in Chinese is expressed either through aspect and/or time adverbials or inferred from context. In this study, we are interested in the interaction of perfective aspect marker -*le* and progressive aspect marker *zai* with ACTs and ACCs. In Chinese, predicates such as *huahua* ‘draw picture(s)’ are ACTs (unbounded and with no natural endpoint) compatible with the imperfective aspect marker *zai*. By contrast, predicates such as *hua yi-fu hua* ‘draw a picture’ are telic ACCs compatible with the perfective marker -*le*. It is important to note that, despite the prototypical association, ACTs can go with -*le*, as -*le* indicates “completion” when it occurs with a verb encoding a telic situation (e.g., resultative verb compound such as *chi-wan* ‘eat-finish’) and it indicates “termination” when occurring with atelic situations (e.g., atelic verbs such as *chi* ‘eat’; Li, 1990). Similarly, *zai* can be associated with most situation types except for (individual) states (? *zai zhidao* ‘is knowing’; Xiao and McEnery, 2004, p. 209). Specifically, when it comes to ACCs, *zai* can occur with “non-completive” ACCs (e.g., *zai hua yi-fu hua* ‘is painting a picture’, *zai kan yi-ben shu* ‘is reading a book’), but not ACCs with a goal, duration, or distance (? *zai zou yi-quan xiaoyuan* ‘walk one round campus’; Liu, 2012, p. 160).

One of the earlier studies on the AH in Chinese SLA was conducted by Jin and Hendriks (2005), who employed a narrative story-telling task to explore learners’ use of *-le*, *zai*, and *-zhe* (a durative marker denoting the imperfective aspect). They reported several pieces of evidence in support of the AH: Their L2 participants used perfective more than imperfective markers at the beginning and both their L2 participants and their control group (L1 adults) used perfective-*le* largely in associations with achievements. Further, an increase in L2 participants’ proficiency level went hand in hand with the decrease of perfective-*le* with achievements, suggesting the spread of perfective to other situation types when learners’ proficiencies increased. Meanwhile, all the tokens of L2 participants’ perfective-*le* with ACT predicates were ungrammatical, suggesting continuous difficulty in non-prototypical associations. For the imperfective marker *zai*, both the native speakers and L2 learners mostly used it with ACT predicates, again conforming to the prototype predictions. Further, while the L1 control group also used *zai* occasionally with ACCs, the L2 group used *zai* with ACCs in ungrammatical ways, indicating challenges in acquiring non-prototypical associations.

A more recent study using production data was Yang (2016), who extracted 60 Chinese essays written by English native speakers across four different proficiency levels from the Chinese Interlanguage Corpus by the Beijing Language and Culture University. Examining learners’ usage of *-le* and *-zhe*, Yang (2016) reported findings that were also generally consistent with the AH, in that perfective-*le* occurred most often with accomplishment and achievement predicates and much less so for activity and state predicates. While both Jin and Hendriks (2005) and Yang (2016) used production data only, Liu (2012) employed a judgment task and a production task based on pictures with L2 participants with English L1 background and found patterns deviating from the AH. For instance, none of the L2 participant subgroups from low to high proficiency levels in Liu (2012) showed statistical differences from L1 Chinese adults’ performance in *zai*’s association with non-completive ACCs (e.g., *kan yiben shu* ‘read a book’); meanwhile, Liu’s low-level (but not intermediate or high-level) participants showed significantly lower accuracies than native speakers in *zai*-ACT associations. The author interpreted this result as evidence that non-completive ACCs was acquired earlier than ACT predicates with *zai* associations by L2 participants. This pattern contradicted the prototypical associations predicted by the AH. While Liu (2012) discussed the role of L1 transfer to account for other findings in her study, transfer did not seem to account for this pattern of deviation from the AH. Another study reporting evidence inconsistent with the AH was Tong and Shirai (2016), who used a written editing judgment task. In their study, L2 learners (2nd and 3rd year learners of Chinese) had to judge whether the use of -*le* and *zai* are obligatory, forbidden, or optional in association with different lexical aspects in sentences and paragraphs. They found that although learners’ use of *zai* conformed to the prototype associations (with *zai* associations with ACT predicates judged as obligatory most of the time), the developmental patterns in learners’ judgment of *zai* and -*le* contradicted the AH: Whereas the AH predicts the acceptance of less-prototypical associations in higher proficiency levels, the higher-level L2 participants in their study showed stronger associations between *zai* and ACTs and stronger associations between -*le* and ACCs than lower-level participants. Whereas Tong and Shirai (2016) used the default past tense hypothesis (Salaberry, 1999) to explain their findings, their results may also be affected by their determination of lexical aspect categories: While their study mentioned the use of diagnostic test developed by Chen and Shirai (2010), some lexical aspect coding, such as *mai* ‘buy’ as an accomplishment verb and *zuo-che* ‘ride-the-bus’ and *wen* ‘ask’ as achievement verbs, may be questionable.

## The present study

The above literature review section shows that task variations (i.e., online tasks that tap into implicit knowledge and offline tasks into explicit knowledge) and the role of individual differences especially in working memory capacity in the L2 processing and acquisition of temporality have often been overlooked in the past. In addition, when it comes to L2 Chinese research on this topic, studies have exhibited variations in their degree of support for the AH.

The goal of this study was to investigate the processing and acquisition of Chinese temporality in L2 learners of Chinese and the effects of grammatical and lexical aspects on these processes. We are interested in the extent to which these effects may be predicted by the AH. To this end, we employed a sentence-picture matching task, which has been argued to be a suitable method for assessing implicit linguistic knowledge (Orfitelli and Polinsky, 2017), to collect data regarding how accurately and quickly learners process temporality online. We also included an offline written translation task, in which learners can draw on explicit knowledge (Loewen, 2009), to generate data regarding how learners comprehend a given linguistic input (Campbell, 2014). With the triangulation of online and offline tasks, and with considerations of potential individual differences in language proficiency and WMC, we aim to provide a more complete picture of L2 learners’ processing and comprehension of temporality. Specifically, we seek to address the following research questions (RQs):

RQ1: How do grammatical and lexical aspects influence the online comprehension of temporal event structures in L2 learners of Mandarin Chinese?

RQ2: How do grammatical and lexical aspects influence the offline comprehension of temporal event structures in L2 learners of Mandarin Chinese?

RQ3: Do learners’ proficiency and working memory capacity modulate these processes? If so, how?

## Materials and methods

### Participants

Thirty-one L1-English students learning Chinese at a university in northeast U.S.A. participated in the experiments (age 18–35, mean age 20.7). They were all enrolled in or had completed second-year, third-year, or fourth-year Chinese courses at the institution. They have learned Chinese for 2.7 years on average (*SD* = 0.8, Range 2–4). For the exploratory analysis of influence from proficiency in this study, L2 proficiency was explored either as a continuous variable or as a categorical variable. The operationalization of proficiency as a continuous variable is detailed in the section on “Data treatment and analysis.” The operationalization of proficiency as a categorical factor was based on participants’ course enrollment or completion. Specifically, participants who were enrolled in or had completed Second Year Chinese II as their highest level Chinese course were considered the first group. As the study was conducted at the end of the semester, those participants had four semesters of classroom instruction; participants who were enrolled in or had completed Third Year Chinese II as their last Chinese course were considered the second group; participants who were enrolled in or had completed Fourth Year Chinese were considered the third group. Based on the annual in-house Oral Proficiency Interviews implemented at the institution, participants in the first group (with four semesters of Chinese learning) had intermediate-mid to intermediate-high level proficiency based on ACTFL standards.^2^ Participants in the second group had advanced-low level proficiency, and participants in the third group had advanced-mid or higher proficiency. For convenience and from here on, these three groups will be, respectively, referred to as the “intermediate” (*n* = 15), “advanced” (*n* = 10), and “advanced-plus” (*n* = 6) groups. In addition, while participants’ language background surveys revealed that their exposure to Chinese was primarily through classroom instruction and English was the dominant language for all participants, fourteen of the L2 participants reported that they have one or two parent(s) who speaks at least one dialect of Chinese. Because those participants were possibly exposed to English and Chinese simultaneously in their home setting, we refer to those 14 participants as heritage language learners, in contrast to non-heritage learners who had no home exposure to Chinese. It should be noted that while 6 of those 14 participants reported both English and Chinese as the first languages that they learned, all 31 participants reported that their exposure to Chinese was primarily through classroom instruction and that English was their dominant language. In consideration of the potential influence of participants’ heritage background in their performance patterns, heritage vs. non-heritage language experience was also probed as an exploratory analysis in this study.

Twenty-nine Chinese native speakers as the control group were recruited from two Chinese universities in Mainland China. They were undergraduate or graduate students at the time of the experiments (age 18–23, mean age: 20.2 years). All participants reported normal or corrected-to-normal vision.

### Materials

Experimental items for the sentence-picture match task (SPMT) consisted of 40 critical items in four conditions constructed by crossing two factors: grammatical aspect (Imperfective vs. Perfective) and lexical aspect (ACT vs. ACC). These items were adapted from Yap et al. (2009), where the sentences were presented in Cantonese. In some cases, the lexical items and sentences were modified to suit the proficiency levels of our L2 participants.^3^ After modification, two intermediate proficiency students who were from the same participant pool but did not take part in the experimental tasks checked all experimental materials to ensure that all the lexical items were familiar to participants. One set of example sentences are illustrated in (1).

1. a. Zhe-ge nanhai zai he niunai. (Imperfective-ACT)
  This-CL^4^ boy ZAI-drink milk.
  ‘This boy was drinking milk.’
  b. Zhe-ge nanhai he le niunai. (Perfective-ACT).
  This-CL boy drink-LE milk.
  ‘This boy drank milk.’
  c. Zhe-ge nvren zai zuo yi-kuai dangao. (Imperfective-ACC).
  This-CL woman ZAI-make one-CL cake.
  ‘This woman was making a (piece of) cake.’
  d. Zhe-ge nvren zuo le yi-kuai dangao. (Perfective-ACC).
  This-CL woman make-LE one-CL cake.
  ‘This woman made a (piece of) cake.’

As illustrated, pairs of sentences, e.g., (1a) vs. (1b), differed minimally in grammatical aspects and the contrast between ACTs and ACCs was realized by using different lexical items.^5^ These critical items were distributed into two lists based on a Latin Square design such that a participant would never encounter the pair of (1a) and (1b) or the pair of (1c) and (1d) at the same time, and each participant read 20 items (five items per condition) in the SPMT and 20 items in the translation task, explained in the “Procedure” section. For the SPMT, 18 distractors similar to the critical items in length and linguistic complexity but with unrelated syntactic structures (e.g., *ba*-, *bei*-structures) were created. The distractors helped to disguise the critical trials, making each list in the SPMT ultimately consist of 38 test sentences, counterbalanced across participants. All items in one list were also completely randomized for presentation order in the SPMT. To minimize participant fatigue, no distractor items were included in the translation task.

### Procedure

The complete experiment included an online sentence-picture matching task, a working memory test, an offline Chinese-to-English translation task, and a language background questionnaire, in this order. This task order was deemed most effective to avoid a repetition effect: Despite a Latin Square design, some test sentences may still repeat across tasks. The sentence-picture matching task needed to precede the translation task because participants’ exposure to each sentence in the online task was brief and they were unlikely to retain clear memories of the sentences read. Similarly, the working memory test was inserted between the two tasks to further minimize any potential repetition effects. A language background questionnaire was administered at the end to minimize a possible fatigue effect in the main tasks. The complete experiment was administered *via* Gorilla Experimental Builder (Anwyl-Irvine et al., 2020).^6^

**Figure 1:**
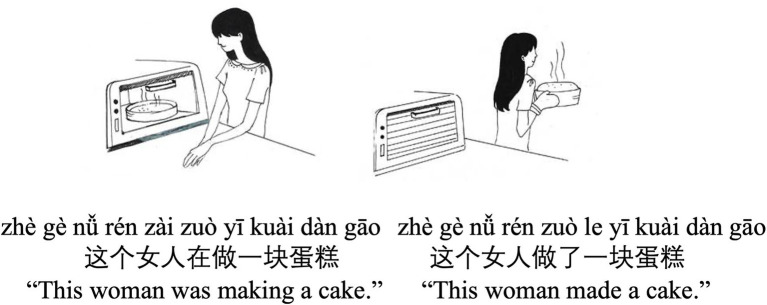
An example of visual stimuli for SPMT.

- *Sentence-picture matching*: Each trial consists of a sentence and two pictures (see Figure 1 for an example).^7^ Every sentence was inter-word spaced and presented with simplified Chinese characters along with *pinyin* (i.e., a Chinese phonetic transcription system) on top of each word.^8^ This was done to facilitate text comprehension for learners of Chinese (Bassetti and Lu, 2016). On a given trial, a fixation cross appeared on the screen for 1 s indicating a sentence to come. The sentence then showed up for a maximum of 15 s for the participant to read through. Subsequently, two black-and-white line-drawn pictures appeared side by side for 8 s on a separate screen, and the participant’s task was to choose the picture that goes with the sentence by pressing the “F” key on the keyboard of the computer if they thought the picture on the left was correct and pressing the “J” key if they thought the picture on the right was correct. The expected answer was counterbalanced for positions such that half of the trials had the answer on the left and the other half on the right. The position of the expected answer across the trials was also randomized to prevent participants from developing certain processing strategies. For both sentences and pictures, they could choose to proceed by pressing the spacebar within the time limit and did not have to wait until the end of the presentation. The presentation timing was determined based on a small pilot study (*n* = 3). A block of five practice trials preceded the main task for participants to familiarize themselves with the task.
- *Working memory test*: A backward digit span task was used to measure general working memory capacity (WMC; Wechsler, 1981). We employed the test because we were interested in the general influence of WMC rather than language-related WMC such as verbal/phonological WMC. Participants saw and recalled the digits (e.g., 2,5,3) presented at a 1 s rate in the reverse order (e.g., 3,5,2) by typing them down in a computerized text box. They were tested on digit sequences of two to seven digits. Two trials for each sequence length were provided, making up a total of 12 trials. The test does not stop until one fails in both trials within each given sequence length. A 2-digit practice trial was provided for participants to familiarize themselves with the task.
- *Chinese-into-English translation task*: Participants were instructed to read and translate 20 critical Chinese sentences into English by typing out the corresponding English sentences. The Chinese sentences were also complemented with *pinyin*. An example Chinese sentence for translation is illustrated in (2).
  zhè gè nán hái zài hē niú nǎi

2. 这个 男孩 在 喝 牛奶。
  - *Background questionnaire*: The background questionnaire for the L2 participants asked for information regarding their age, their native language, onset age of learning Chinese, length of Chinese learning, Chinese courses enrolled, experience residing in a Chinese-speaking country or region, and self-reports on four of the language skills (listening, speaking, reading, and writing) on a 5-point Likert Scale. For Chinese L1 participants, the only biographical information collected was their age and native language.

### Data treatment and analysis

All statistical analyses were conducted with the R programming language (R Core Team, 2021). The ‘tidyverse’ package version 1.3.1 was used for visualization (Wickham et al., 2019). The ‘plot_model’ function from the ‘sjPlot’ package version 2.8.9 was used to plot the model fit (Lüdecke, 2021). With the package ‘lme4’ version 1.1.27.1 (Bates et al., 2015), data collected from each task were analyzed following the mixed-effects modeling procedures to take into account the variation in participants and stimuli at the same time (Linck and Cunnings, 2015). Data and analysis code are publicly available on the OSF website.^9^

In the spirit of the Bilingual Turn (Ortega, 2009),^10^ we did not compare the performance of L2 learners and that of native speakers. Rather, taking learner grammar as a system of its own (Cook, 2008), we focus on the contrasts across within-subject conditions among learners (e.g., differences in RTs between perfectives and imperfectives). Data were analyzed and reported with a focus on the L2 group.

In general, three sets of data were analyzed: (1) Accuracy rates from the SPMT, (2) Response times (RTs) from SPMT, and (3) Accuracy rates from the translation task. We analyzed RTs from accurate trials only to rule out the potential influence of speed-accuracy trade-offs on RTs in the sense that participants were likely to respond more slowly when they responded more accurately. RTs from SPMT were log (natural logarithm) transformed to adjust for the skewness of the data distribution. Plots of model residuals against fitted values and Q-Q plots based on log RTs revealed no obvious deviations from normality and homoscedasticity. Participants’ translations were coded in a binary fashion as either *correct* or *incorrect* for the grammatical aspect (GA-correct, GA-incorrect) and the lexical aspect (LA-correct vs. LA-incorrect) (where ‘correct’ means the correct use of perfective or imperfective aspect and the correct use of ACTs or ACCs in Chinese) in the sentence. Accuracy was not coded for linguistic features unrelated to key areas (i.e., GA and LA).

The coding task was done by two trained Chinese native speakers who are highly proficient in English. One speaker served as the primary coder and the other as the secondary coder. Cohen’s Kappa was used to assess inter-rater reliability and was calculated for the coding of the grammatical aspect and the lexical aspect in each group. Cohen’s Kappa indicated that participants’ accuracy rates on the translation task were substantially agreed upon across the board for coding between coders (Cohen’s Kappa for L1-GA: 0.73, *p* < 0.001; L1-LA: 0.7, *p* < 0.001; L2-GA: 0.86, *p* < 0.001; L2-LA: 0.82, *p* < 0.001), as interpreted based on Cohen (1960). Data from the primary coder were submitted to statistical analysis. Working memory score was calculated out of 12 (1 point per trial * 12 trials in total). Data points for WMC were trimmed in a way that the times taken to respond to each trial beyond 2.5 standard deviations (SDs) from the mean by participant were removed, affecting less than 2% of the data for either group.

To operationalize proficiency as a continuous variable, the overall proficiency score was derived through a Principal Component Analysis over six different numerical measures (centered and scaled): total years of learning, semesters of college Chinese courses taken, self-rated proficiency in listening (*M* = 3.5, *SD* = 1, Range 2–5), speaking (*M* = 3.4, *SD* = 0.88, Range 2–5), reading (*M* = 3.4, *SD* = 0.9, Range 2–5), writing (*M* = 2.7, *SD* = 1, Range 2–5). The first component explains the largest amount of variation (36%) and was therefore used to approach the overall proficiency for participants. The factor loadings for the first component on each measure (rotated factor solution) were − 0.01 for years of learning, 0.50 for semesters of learning, −0.53 for listening, −0.24 for speaking, 0.52 for reading, 0.38 for writing.

With respect to statistical modeling, continuous RTs data were fit using linear mixed-effects models (Baayen et al., 2008) and accuracy rate data with a binomial distribution were fit using logistic mixed-effects models (Jaeger, 2008). Fixed effects were grammatical aspect and lexical aspect in statistical models set up for both groups. The L2 group was also assessed for the influence of language proficiency, WMC, and language exposure experience as additional fixed effects that were included in relevant models. We also added sentence length (centered and scaled) and its interactions with other predictors as covariates to account for its potential influence.^11^ Sum-coding (−0.5, 0.5) was adopted for categorical predictors to obtain ANOVA-style main effects and interactions.^12^ *Post-hoc* tests were performed using the ‘emmeans’ package version 1.7.0 (Lenth, 2020), with Tukey adjustments for pairwise comparisons. The random effects structure was kept maximal for the initial model allowed by the experimental design, for which we included by-participant and by-item intercepts, by-participant random slopes for within-subject factors (e.g., grammatical aspect and lexical aspect) and their interactions, and by-item random slopes for between-subject factors (e.g., proficiency, WMC). In cases where models failed to converge, we simplified the random effects structures by iteratively removing the correlation between random effects and the random effect contributing to the least variance until models converged.

## Results

We first report the results from the sentence-picture matching task and then from the translation task. For the SPMT, results of accuracy rates and response times are presented in order. The translation task is reported for the accuracy rates only. Results from the L1 and L2 groups are reported separately. Exploratory analyses were conducted on the L2 data for influences of individual differences including learners’ language proficiency and WMC. As our participants had mixed language exposure experience, participants’ heritage vs. non-heritage background was also considered in our analyses.

## Sentence-picture matching task

### Accuracy rate data

**Figure 2:**
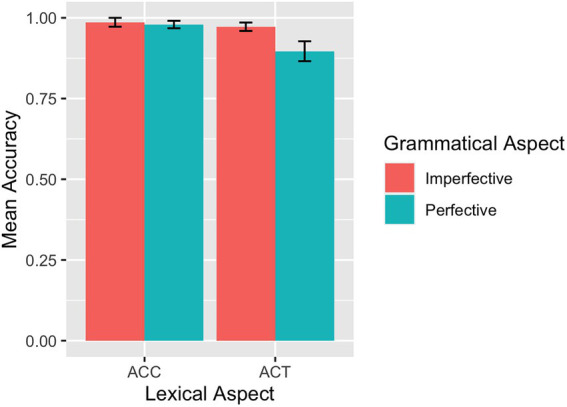
SPMT: Mean accuracy rates in matching sentences and pictures by L1 speakers.

**Figure 3:**
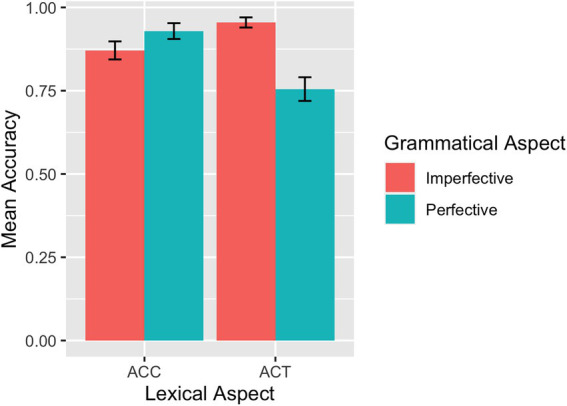
SPMT: Mean accuracy rates in matching sentences and pictures by L2 learners.

The mean accuracy rates across conditions for each group are summarized in Table 1. The descriptive results of the accuracy rates are also visualized for the L1 group in Figure 2 and the L2 group in Figure 3. For the L1 group, the overall mean accuracy rate in matching the picture with the sentence read was 0.96 (*SD* = 0.83). As shown in Table 1 and Figure 2, the L1 group performed at or close to ceiling on ACCs. For the L2 participants, the overall mean accuracy rate in matching sentences and pictures was 0.88 (*SE* = 1.32).

**Table 1:** Response accuracy rates across conditions by group: mean (standard deviation: sd).

Group	ACC		ACT	
	Imperfective	Perfective	Imperfective	Perfective
L1 (*n* = 29)	0.99 (0.07)	0.98 (0.06)	0.97 (0.07)	0.90 (0.17)
L2 (*n* = 31)	0.87 (0.15)	0.93 (0.13)	0.95 (0.09)	0.75 (0.20)

Results of the model fit to the L1 accuracy rate data revealed a significant main effect of grammatical aspect (*β* = −1.23, *SE* = 0.55, *p* = 0.025), indicating that imperfective sentences were responded to more accurately than perfective sentences. Although we did not find a significant interaction between grammatical and lexical aspects, we nevertheless conducted separate analyses to pinpoint the potential locus of the grammatical and lexical aspect effects precisely. Only the model for the ACT sentences showed a main effect of grammatical aspect (*β* = −1.47, *SE* = 0.59, *p* = 0.013) in that imperfective sentences were responded to more accurately than perfective sentences in the case of ACT sentences. Turning to the results for the accuracy rate from the L2 group, the model fit showed a main effect of grammatical aspect (*β* = −0.64, *SE* = 0.29, *p* = 0.029), induced by higher accuracy rates for imperfective sentences than for perfective sentences. In addition, there was an interaction between grammatical aspect and lexical aspect (*β* = −2.62, *SE* = 0.59, *p* < 0.001): Imperfective sentences were responded to with higher accuracies than perfective sentences if they contain ACTs (*β* = 1.95, *SE* = 0.43, *p* < 0.001) and perfective sentences were responded to with marginally higher accuracies than imperfective sentences if they contain ACCs (*β* = −0.67, *SE* = 0.40, *p* = 0.091).

When included in the model as a continuous variable, language proficiency did not exhibit any effect. Language proficiency was then added to the model as a categorical variable and again showed no main effect and interaction between grammatical aspect and lexical aspect. However, treating proficiency as a categorical predictor allowed us to conduct by-proficiency level analyses to detect if each level of L2 participants revealed the same pattern of effects caused by the grammatical and lexical aspects. For the intermediate level L2 subgroup, a significant interaction between grammatical aspect and lexical aspect was observed (*β* = −2.94, *SE* = 1.05, *p* = 0.005) and this L2 subgroup had significantly more accuracies on imperfective sentences than on perfective sentences in the ACT condition (*M* = 0.96 vs. *M* = 0.76). The same imperfective advantage in ACT condition was observed among both the advanced level (*M* = 0.92 vs. *M* = 0.78) and the advanced-plus level subgroups (*M* = 1.0 vs. *M* = 0.7). In other words, in all the three proficiency subgroups, imperfective sentences were responded to more accurately than perfective sentences in the ACT condition.

**Figure 4:**
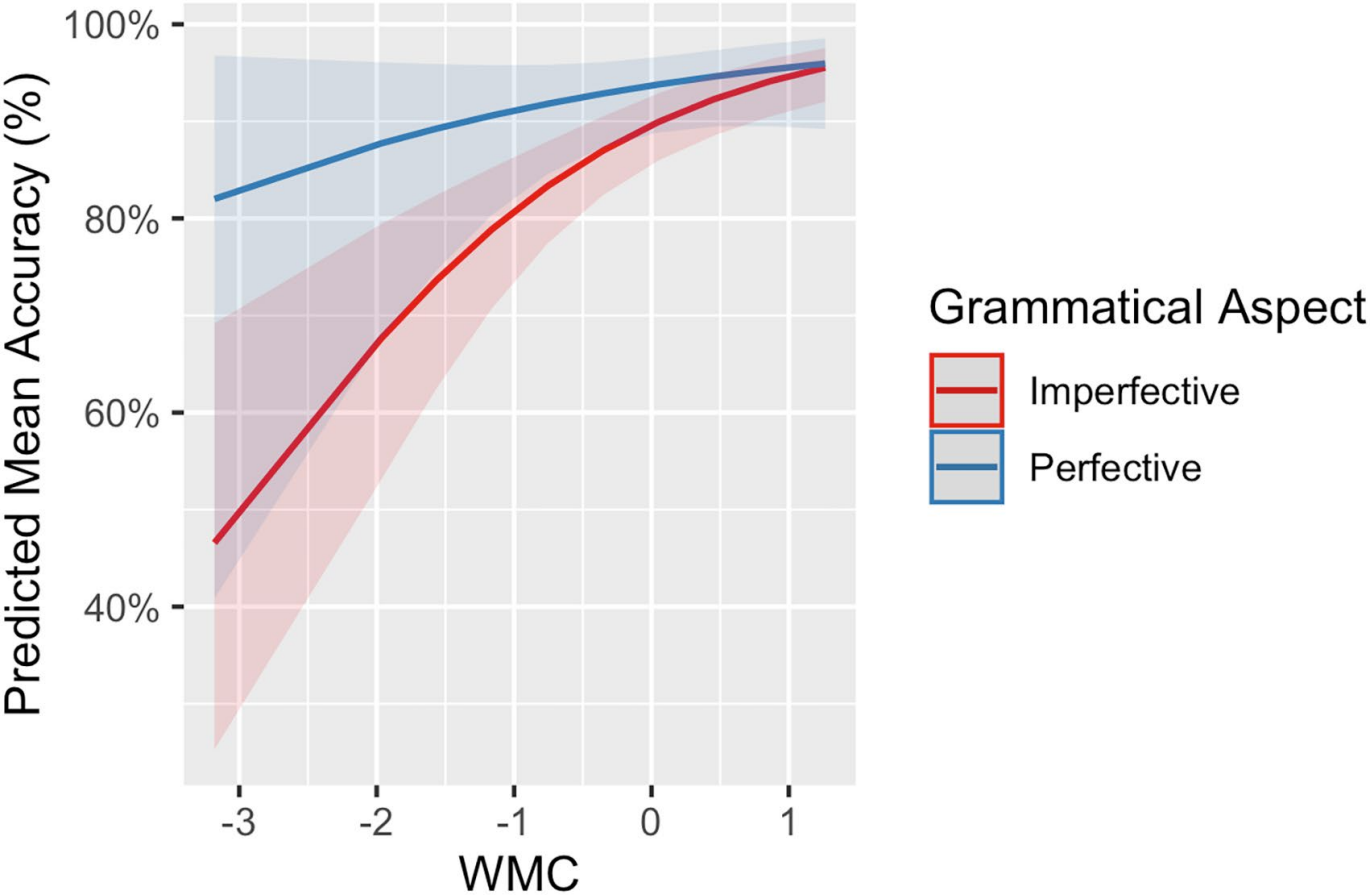
SPMT: The difference in mean accuracy rates between imperfectives and perfectives as a function of WMC in L2 learners.

The influence of WMC on L2 participants’ accuracy rates was also explored. The model for the L2 group including working memory span (centered and scaled) and its interaction with grammatical aspect and lexical aspect returned a main effect of working memory span (*β* = 0.33, *SE* = 0.16, *p* = 0.035). That is, not surprisingly, participants with a higher working memory span responded to the stimuli more accurately. More crucially, grammatical aspect significantly interacted with working memory (*β* = −0.56, *SE* = 0.28, *p* = 0.046). Mean accuracy rates for each grammatical aspect (imperfective vs. perfective) are presented as a function of L2 participants’ working memory scores in Figure 4, which were plotted from the model fit. For both imperfective and perfective aspects, L2 participants with higher WMC performed more accurately than those with lower WMC. As accuracies in imperfectives improved more rapidly than accuracies in perfectives, the difference in accuracy rates between imperfectives and perfectives decreased as participants’ WMC increased.

Finally, we probed the potential influence of participants’ heritage vs. non-heritage language experience by modeling participants’ accuracy rates as a function of language exposure experience (heritage vs. non-heritage) and its interaction with the grammatical and lexical aspects. The model fit revealed no main effect and interaction for language experience. Separate analyses on heritage and non-heritage language participants showed that both groups of participants responded to imperfective sentences more accurately than to perfective sentences in the case of ACT (Heritage: *β* = 2.07, *SE* = 0.66, *p* = 0.002; Non-heritage: *β* = 1.88, *SE* = 0.57, *p* = 0.001).

### Response time data

**Figure 5:**
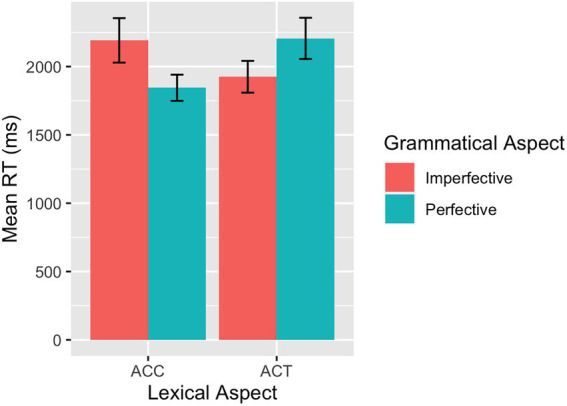
SPMT: Mean RTs across conditions in L1 speakers.

**Figure 6:**
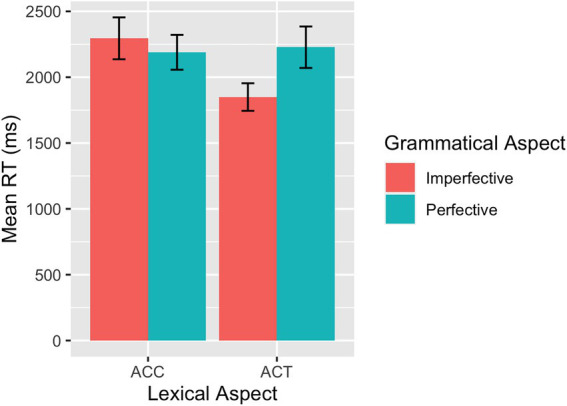
SPMT: Mean RTs across conditions in L2 learners.

Descriptive statistics for raw response times by group are reported in Table 2 and plotted for the L1 group in Figure 5 and for the L2 group in Figure 6.

**Table 2:** Response times across conditions by group: mean (standard deviation: sd).

Group	ACC		ACT	
	Imperfective	Perfective	Imperfective	Perfective
L1 (*n* = 29)	2191.57 (876.50)	1844.68 (515.57)	1925.19 (626.25)	2206.23 (811.04)
L2 (*n* = 31)	2295.10 (885.34)	2188.93 (737.12)	1849.26 (582.61)	2227.70 (877.28)

The model for the RT data from the L1 group showed a significant interaction between grammatical aspect and lexical aspect (*β* = 0.29, *SE* = 0.06, *p* < 0.001), driven by shorter RTs for perfectives than for imperfectives in the ACC condition and shorter RTs for imperfectives than for perfectives in the ACT condition, as confirmed in the *post hoc* pairwise comparison. Results of the model fit to the data from the L2 group demonstrated a main effect of grammatical aspect (*β* = 0.07, *SE* = 0.03, *p* = 0.025), indicating that imperfective sentences overall were processed faster than perfective sentences. The interaction between grammatical aspect and lexical aspect was significant (*β* = 0.15, *SE* = 0.07, *p* = 0.030) and ACT sentences were processed faster for imperfectives than for perfectives.

The influence of language proficiency, WMC, and language exposure experience on the L2 performance was evaluated in order. First, if treated as a continuous variable, language proficiency neither surfaced as a main effect nor interacted with any other fixed factor (grammatical aspect and lexical aspect). If treated as a categorical variable, proficiency level did not modulate RTs. As has been done for the accuracy rate data, separate analyses by proficiency level were conducted to precisely locate the effects of the grammatical and lexical aspects within each proficiency level. Specifically, intermediate level participants were shown to process imperfective sentences faster than perfective sentences in the case of ACT (*β* = −0.16, *SE* = 0.07, *p* = 0.029). For advanced level participants, RTs for imperfective sentences were not significantly different from those for perfective sentences. Advanced-plus level participants, on the other hand, showed a pattern that was similar to intermediate level participants in that they processed imperfective sentences with ACTs significantly faster than perfective sentences with ACTs (*β* = −0.25, *SE* = 0.11, *p* = 0.019).

**Figure 7:**
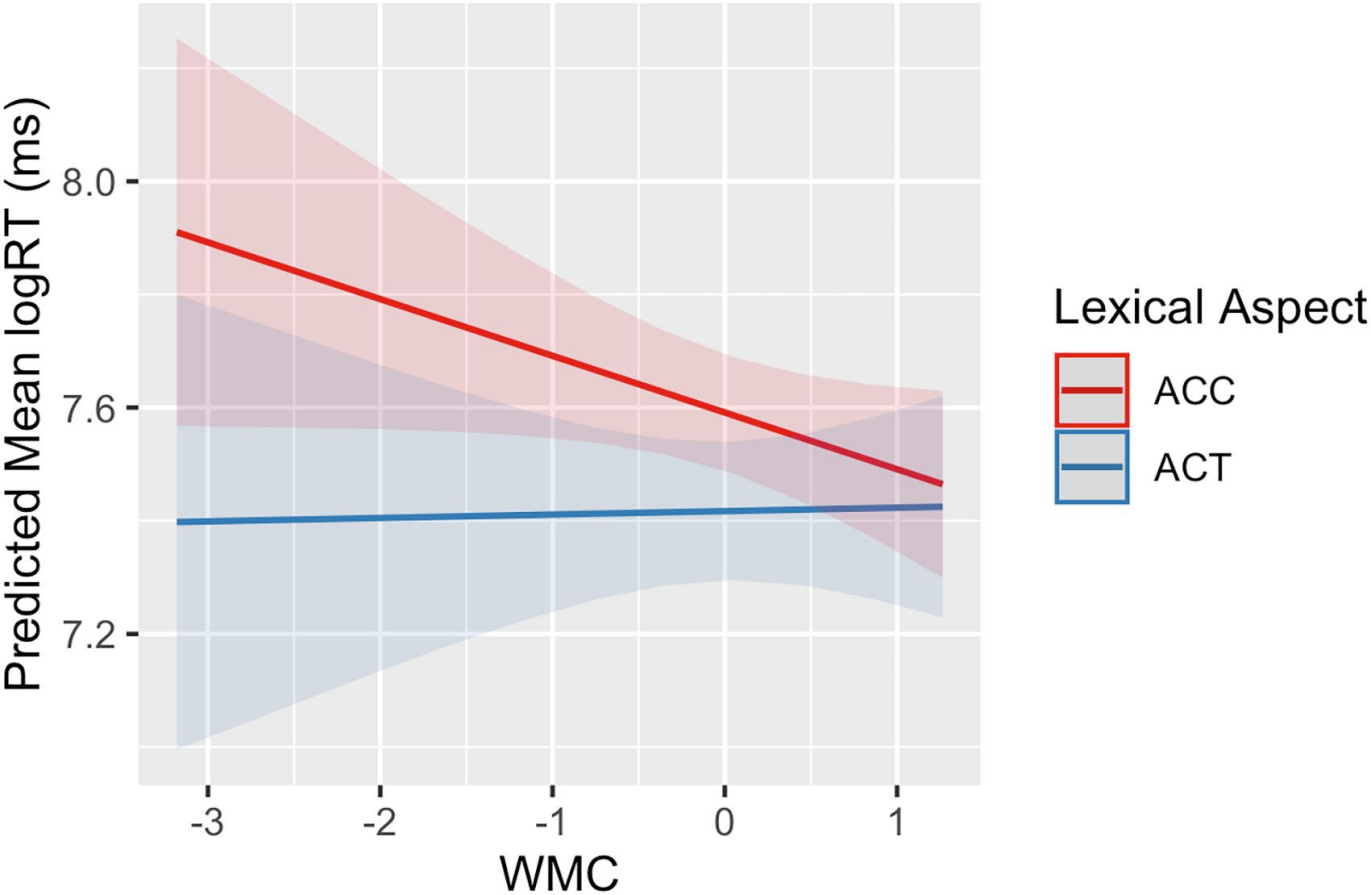
SPMT: The difference in mean RTs between ACTs and ACCs as a function of WMC in L2 learners.

Second, WMC was explored for its effects on the processing performance of L2 participants. Results from the model including working memory and its interaction with the grammatical and lexical aspects showed an interaction between working memory and lexical aspect (*β* = 0.09, *SE* = 0.03, *p* = 0.005). Figure 7 illustrates the size of the difference in RTs between ACTs and ACCs as a function of L2 participants’ working memory capacity. RTs especially for ACC verbs decreased as participants’ WMC increased. As a whole, it showed that the difference in RTs between ACT and ACCs decreased as participants’ WMC increased.

Third, modeling RT data by L2 participants as a function of language experience and its interaction with the grammatical and lexical aspect yielded no main effect or interaction for language experience. We nevertheless conducted separate analyses on heritage and non-heritage language participants. It was found that heritage language participants processed imperfective sentences faster than perfective sentences in the case of ACTs (*β* = −0.21, *SE* = 0.07, *p* = 0.001), but there were no other significant results.

### Translation task

**Figure 8:**
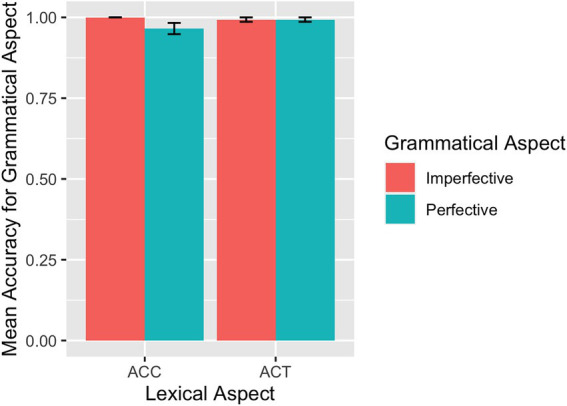
Translation: Mean accuracy rates in translating grammatical aspect by L1 speakers.

**Figure 9:**
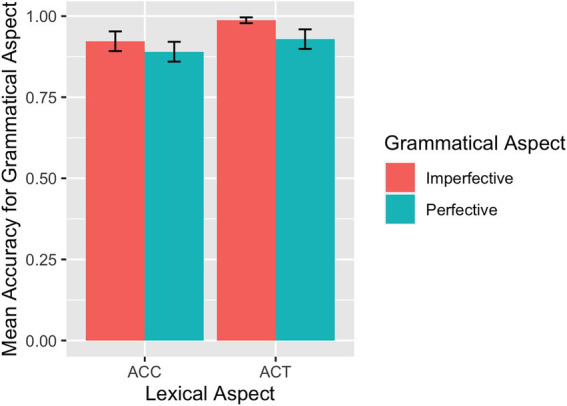
Translation: Mean accuracy rates in translating grammatical aspect by L2 learners.

Table 3 summarizes the descriptive statistics for the accuracy rates across conditions on the translation of grammatical aspect and lexical aspects by each language group. Figure 8 visualizes the L1 group data and Figure 9 the L2 group data on their performance on grammatical aspect translation. The overall accuracy of the L1 participants on the translation of grammatical aspect was 0.99 (*SD* = 0.45), indicating that they performed at or near ceiling in all conditions in the case of grammatical aspect. Since there was not enough variation in the accuracy rates for any relationship to be detected, we did not conduct any statistical modeling in this case.

**Table 3:** Translation accuracy rates across conditions by group: mean (standard deviation: sd).

Aspect	Group	ACC		ACT	
		Imperfective	Perfective	Imperfective	Perfective
Grammatical Aspect	L1 (*n* = 29)	1.00 (0)	0.97 (0.09)	0.99 (0.04)	0.99 (0.04)
	L2 (*n* = 31)	0.92 (0.17)	0.89 (0.17)	0.99 (0.05)	0.93 (0.17)
Lexical Aspect	L1 (*n* = 29)	0.96 (0.08)	0.99 (0.04)	0.97 (0.08)	0.92 (0.10)
	L2 (*n* = 31)	0.92 (0.16)	0.88 (0.13)	0.99 (0.05)	0.92 (0.11)

**Figure 10:**
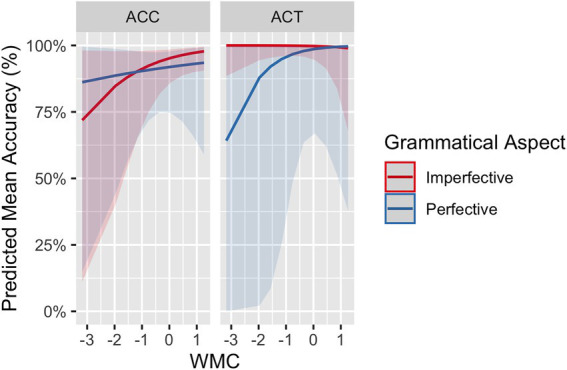
Translation: The difference in mean accuracies in translating grammatical aspect across conditions as a function of WMC by L2 learners.

The overall accuracy of the L2 participants on the translation of grammatical aspect was 0.93 (*SE* = 1.01). Results from the model for the translation of grammatical aspect showed a main effect of grammatical aspect (*β* = −1.20, *SE* = 0.46, *p* = 0.009), driven by higher accuracy rates for imperfective sentences than for perfective sentences. The model also returned a marginal interaction between grammatical aspect and lexical aspect (*β* = −1.58, *SE* = 0.92, *p* = 0.088), indicating that imperfective sentences were translated more accurately than perfective sentences in the case of ACT. Influence from language proficiency (continuous or categorical), working memory, and language experience was explored. No effects of language proficiency and language exposure experience were observed. WMC was found to play some role, as reflected in a marginal three-way interaction among working memory, grammatical aspect, and lexical aspect (*β* = 2.81, *SE* = 1.46, *p* = 0.053). As in Figure 10, for ACCs in both perfective and imperfective aspects, higher WMC was associated with higher accuracy in general. As WMC increases, accuracies in imperfective sentences with ACCs improved more rapidly than accuracies in perfective sentences with ACCs. In other words, WMC affected ACCs in imperfectives more than ACCs in perfectives. For ACTs predicates, ACTs in imperfective received accuracies close to 100% regardless of WMC, while ACTs in perfectives improves as WMC increases.

**Figure 11:**
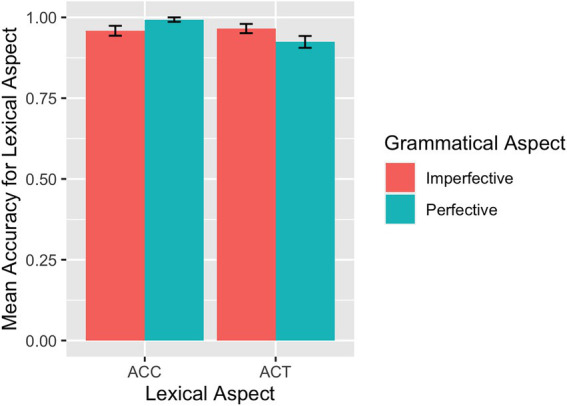
Translation: Mean accuracy rates in translating lexical aspect by L1 speakers.

**Figure 12:**
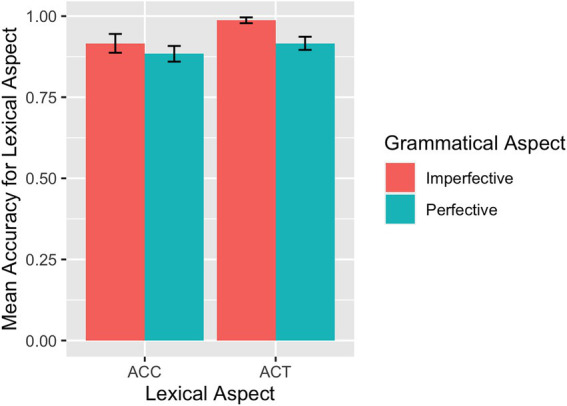
Translation: Mean accuracy rates in translating lexical aspect by L2 learners.

The mean accuracy rate on the translation of lexical aspect is visualized in Figure 11 for the L1 group and in Figure 12 for the L2 group. The overall accuracy of the L1 participants on the translation of lexical aspect was 0.96 (*SD* = 0.81). The overall accuracy of the L2 participants on the translation of lexical aspect was 0.93 (*SE* = 1.05). Modeling accuracy rates on the translation of lexical aspect in the L1 group yielded a marginal interaction between grammatical aspect and lexical aspect (*β* = −4.26, *SE* = 2.30, *p* = 0.064), with higher accuracy rates for perfective sentences than for imperfective sentences in the case of ACCs. Results from the model fit to accuracy rates on the translation of lexical aspect in the L2 group showed a main effect of grammatical aspect (*β* = −1.16, *SE* = 0.43, *p* = 0.007), and imperfective sentences overall received higher accuracy rates than perfective sentences for their translation. The same model also returned a marginal interaction between grammatical aspect and lexical aspect (*β* = −1.60, *SE* = 0.86, *p* = 0.065), driven by higher accuracy rates for imperfective sentences than for perfective sentences in the case of ACT. No apparent effects of language proficiency, working memory, and language exposure experience were detected on the translation of lexical aspect among the L2 participants.

## Discussion

The results of the SPMT suggested that native speakers overall achieved higher accuracies for imperfectives than for perfectives, and this trend has also been observed in L2 accuracy rates for the SPMT and the translation task. Likewise, the main effect of grammatical aspect was evident in L2 RTs from the SPMT, signaling that imperfectives in general were processed faster than perfectives. This result contrasts with the finding from Madden and Zwaan (2003), which used a similar experimental paradigm (i.e., picture verification task) but found a perfective facilitation effect. But the current finding is in line with Magliano and Schleich (2000), which used a quite different method (i.e., spoken narrative task) and obtained a similar imperfective advantage in processing. We consider and evaluate three possible reasons. First, -*le* is relatively more complex than *zai*, both syntactically and semantically (Jing-Schmidt et al., 2022). For example, as discussed earlier, -*le* allows a wider range of semantic interpretation depending on the kind of lexical aspect associated with, and the pragmatic context it occurs. According to the Semantic Complexity Hypothesis (Van Hout, 2008, p. 1753), semantic complexity of aspectual morphemes is consequential in the acquisition of tense-aspect among children, namely semantics of simple semantic operations is acquired early. As such, perfectives are likely harder to process and acquire for learners. This explanation coincides with Duff and Li’s (2002, p. 446) observation that learner difficulties with -*le* can be related to the “multifunctionality” of the aspect marker. Second, the fact that *zai* occurs preverbally and -*le* postverbally may have led to the observed imperfective facilitation. When integrating information from different parts of the sentence during reading, the parser would encounter the aspect marker first and then the verb in the case of *zai*, thus immediately constructing the representation of an ongoing event due to this priming effect. In the case of -*le*, the parser must encounter the verb first and integrate information from the verb processed to the subsequent experienced-*le*, which presumably would take longer for relevant representations of structural events to be built up. In an earlier study by Fang and Yuan (2021), the researchers found that sentences containing the perfective-*le* were processed faster than sentences containing the postverbal-*zhe* at the spillover region in a self-paced study. Thus, the location of the marker may be an additional factor contributing to the difference in RTs in the SPMT. A third possibility is that the activation level for imperfectives decays at a slower rate than that for perfectives, leading to an imperfective advantage, as suggested by Magliano and Schleich (2000).

We now turn to the interactive effects of grammatical and lexical aspects with reference to the prototypical association between certain aspects of L2 processing and acquisition of Chinese temporality, as stipulated in the AH. Cases showing clear ceiling effects were not discussed here because the null effect was likely to be masked by the ceiling effects. In the online comprehension task (SPMT), L1 participants’ accuracies with accomplishments were close to ceiling, thus revealing no significant differences between imperfectives (99% correct) and perfectives (98% correct). Meanwhile, the L1 participants’ data were generally in line with the prototype associations in the AH: L1 participants had faster and more accurate processing with imperfectives than with perfectives in the case of activities, and sentences with ACCs were processed faster for perfectives than for imperfectives. For L2 participants, the prototype effect was reflected in shorter RTs and higher accuracies in ACTs for imperfectives than for perfectives, and marginally higher accuracies in ACCs for perfectives than for imperfectives. Despite no significant difference, RTs in ACCs were also numerically shorter for perfectives than for imperfectives.

In the offline translation task, except for the (near) ceiling effects among the L1 group on grammatical aspect, the prototypical influence emerged from both L1 and L2 groups. For L1 participants, sentences with ACCs were translated slightly more accurately for perfective sentences than for imperfective sentences. For the L2 participants, sentences with ACTs were translated more accurately for imperfectives than for perfectives. Thus, the results of the online and offline tasks in both groups of participants generally follow the prototype predictions. As online tasks are believed to tap into learner’s implicit knowledge and offline data can reflect explicit knowledge, this also shows that participants’ implicit and explicit knowledge converge in their learning of the grammatical and lexical aspects. This prototype effect is in agreement with previous L2 Chinese studies using production data (Jin and Hendriks, 2005; Yang, 2016). Because the pattern was consistent across tasks and in both L1 and L2 data in the study, our results indicate that the prototypical association as a fundamental principle guides the cognitive processing among language speakers in general. This result can also be considered an extension of Yap et al. (2009), who used the SPMT among Cantonese speakers and made similar arguments. As mentioned earlier, the AH can be explained from the angle of semantic congruency (Anderson, 1993). According to Shirai (2010), the grammatical and lexical association due to semantic congruency is closely related to input frequency, as “…the semantic bias comes from biased frequency distribution in the input…” (p. 186). As such, certain combinations should be statistically more frequent than others in the language input. Specific to Chinese, based on Xiao and McEnery’s (2004, p. 104-105) analysis of 1,138 samples in a Chinese corpus, the distribution of perfective-*le* used with the four main predicate types are as follows: achievement 49.9%; accomplishment 29.6%; activity 13.1%; state 5%. When it comes to the imperfective *zai*, it occurs predominantly with ACTs: Out of a total sample of 88 *zai* in the corpus reported by Xiao and McEnery (2004, p. 209), 73 tokens occur with ACTs and 8 and 3 tokens with accomplishments and achievements, respectively. In other words, the frequency distributions map onto the prototype associations specified in the AH. As the present study found prototypical associations in both L1 and L2 data, results indicate that language learners can be sensitive to such distributional statistics.

For the L2 group, the prototypical association was found clearly in SPMT accuracies for ACT predicates and marginally for ACC predicates, and the prototypical preference pattern was also more evident in ACTs compared to ACCs in RT data (i.e., significant differences between grammatical aspects in ACTs but only non-significant numerical differences in ACCs). Similarly, the prototypical advantage only showed up in the case of ACTs but not ACCs in the translation task for L2 participants. In other words, for the L2 participants in our study, Chinese ACTs appear to be more robust than ACCs in their prototypical association with relevant grammatical aspect markers. We believe that the input frequencies of bounded events in Chinese and the association of perfective marker -*le* with various predicates are responsible for this pattern. Xiao and McEnery’s (2004) data, cited above, showed that the perfective aspect occurs more often with achievements than with accomplishments. Similarly, Yang (2016) argued for an associative hierarchy between various predicates and perfective-*le*, with achievements being the most associative, followed by ACCs, and with ACT and stative predicates being the least associative. The Chinese perfective aspect, aside from being associated with [+telic], is often associated with [−durative] or [+punctual] (e.g., Yang, 2016). Thus, ACC predicates are not the most prototypical predicate that goes with -*le*. This could explain why the prototype preference showed up more evidently in the ACT cases than in ACCs, since *zai* statistically occurs with ACTs most among the four predicate types. It should be noted that the lack of a clearer contrast between grammatical aspect preferences in ACCs was observed primarily in the L2 group. We contend that this could still be the result of input frequencies. In language classrooms where L2 participants receive most of their language exposure, teachers most likely drill students with different verbs in combination with event structures. These verbs taught and drilled as vocabulary items can be activity, state, or sometimes accomplishment verbs (such as resultative verb compounds). Accomplishments in Chinese, however, are unlikely to be expressed in a single vocabulary item and more often take the form of verbs plus additional elements, such as verbs + quantified noun phrases (e.g., *kan yi-ben shu* ‘read a book’), verbs plus durational or distance phrases (e.g., *pao liang xiaoshi* ‘run two hours’; *pao yiquan* ‘run a circle’) or verbs taking a goal (e.g., *fei qu Beijing* ‘fly to Beijing’). When L2 students learn grammatical aspects in the classroom, they may be exposed to a variety of verb associations with different aspect markers and ACCs can come up less, as instructors often aim to provide students with richer varieties of vocabulary items (e.g., *da qiu* ‘play ballgames’; *chi-fan* ‘have meal; eat’; *kan dianying* ‘watch movies’) instead of repeatedly using the same verbs to create different ACCs (e.g., *he yi-bei shui ‘drink a cup of water’*; *he yi-ping kele ‘drink a bottle of cola’*). This practice of using more ACT verbs in the classroom was confirmed in our follow-up interview with the instructors who taught our participants. That is, assuming that-*le* occurs with ACCs about 29.6% of the time in L1 speakers’ language exposure, -*le* may occur with ACCs much less in the L2 learners’ language input and output. This input frequency explanation is consistent with several earlier studies that pointed to a usage frequency effect (e.g., Wen, 1997) or the factor of learners’ exposure to typical constructions that co-occur with -*le* (e.g., Duff and Li, 2002). This biased pattern of prototype effect had also been reported in previous L2 studies using production data. For instance, in Yang’s (2016) analysis of L2 Chinese learners’ production in a composition corpus, 39% of the achievement predicates constitute *le*-obligatory context and learners supplied the aspect marker 90% of the time, whereas 30% ACC predicates required-le and learners supplied the marker 87% of the time. While no statistical significance can be claimed regarding the difference, the pattern indicated that learners associated perfective -*le* more with achievements than with accomplishments. In addition, Jin and Hendriks (2005) found that ACCs were used together with their prototypical perfective aspect marker -*le* to a much lesser extent than activities together with their prototypical imperfective aspect marker *zai* in both native speakers and learners. Overall, among Chinese L2 learners, the association between ACTs and imperfective aspect is consistently observed across tasks in different studies, whereas the association between ACCs and perfective may be of a less degree in strength.

Finally, we turn to the influence of learners’ heritage/non-heritage experience, L2 proficiency, and WMC. The only effect of heritage language exposure experience arose from the RTs data such that only the heritage language speakers but not the non-heritage group processed the imperfective sentences faster than perfective sentences in the case of activities. We tentatively suggest that language input from home versus from a formal classroom setting may lead to different acquisition patterns. The impact of the language exposure variable often went unaddressed in the previous literature. Our findings point to the need to scrutinize the potential influence of heritage language exposure on the AH predictions, but due to our small sample size, no conclusive interpretation is possible at this stage.

Next, while there was no effect of language proficiency when it was treated as a continuous variable, possibly due to the relatively narrow range of proficiencies included, some interesting patterns emerged when proficiency was treated as a categorical variable: Learners at the intermediate and the advanced-plus level, but not at the advanced level, processed sentences with ACTs faster for imperfectives than for perfectives. This result suggests that prototypical patterns did not necessarily develop linearly as predicted by the AH; rather, in some cases, the development of grammatical and lexical associations can be U-shaped or reverse U-shaped (e.g., Salaberry, 1999; Ryu et al., 2015). For example, in Salaberry’s (1999) study, a greater non-prototypical effect showed first, and then disappeared, and showed again, as learners’ proficiency increased. In McManus (2013), the author also suggested that a U-shaped development might explain why their prototypical association was not found in their low-proficiency group but in their advanced-proficiency group. Our study found that learners from proficiency levels at both ends within the investigated range exhibited a prototypical effect in both accuracies and reaction times in the sentence-picture matching task, but those from the mid-proficiency level group did not exhibit such effects as consistently. One conceivable explanation is that our advanced-plus group was still in their developmental paths to overcome the constraint of the prototype effect. Another possibility is that the advanced-plus group, similar to the L1 participants in this study, performed in a pattern that was affected by the Semantic Congruency Principle or input frequency. Note that the U-shaped pattern was only observed in ACT predicates in reaction times in SPMT but not in ACC predicates or in other tasks, and the exact shape of learners’ developmental path crossing various predicates and wider proficiency levels is worth investigating in future research.

Compared to L2 proficiency, WMC seems to be more consistent and robust in its effect, because it arose across different tasks. The results of the RTs and accuracy data from the SPMT suggested that learners with higher WMC tend to process the aspectual information faster and more accurately. These results are in line with findings from many previous studies on the positive role of WMC in L2 sentence processing (see Roberts, 2012, for a comprehensive review). It is reasonable to assume that cognitive resources afforded by WMC facilitate language processing, especially in the case of L2 processing, which taxes memory resources more than L1 processing. Therefore, learners with high WMC generally performed well across different grammatical aspects and lexical aspects. The modulating effect of WMC helps decrease processing cost differences (either RT or accuracies) between the two grammatical aspects and also between the two lexical aspects. Particularly interesting is the observed WMC effect for the translation of grammatical aspect, signified by a three-way interaction among grammatical aspect, lexical aspect, and WMC. As in the SPMT, WMC contributed to L2 performance: Learners with higher WMC performed better than those with lower WMC, evident in perfective sentences with both ACCs and ACTs and in imperfective sentences with ACCs. The effect of WMC, however, did not surface for imperfective sentences with ACTs due to the ceiling effect. To the best of our knowledge, this is the first study that explored and detected the influence of WMC in both the online and offline tasks examining the L2 processing and acquisition of temporality. We propose that individual differences in WMC should be modeled as a factor among others to account for learners’ aspect acquisition and processing profiles.

From the perspective of teaching and learning, the prototype effect revealed in this study can help instructors understand why associations between aspect markers and specific predicate types may be easier for learners and how learners gradually extend the use of aspect markers to non-prototypical association. Further, our suggestion regarding the effect of input frequency in learners’ exposure also has several implications to practice. For instance, when a new aspect marker is first introduced, instructors can use pedagogical materials that highlight the salient semantic features of aspect markers (e.g., the boundedness of *-le*) by frequently using them with lexical items and constructions with congruent features (e.g., resultative verb compounds and accomplishment predicates taking the “verb + quantified noun phrase” forms). When learners have acquired these prototypical associations, it can then be beneficial for instructors to expose them to a variety of predicate types to overcome the prototype constraint. Second, findings from the study suggest that intermediate level learners still need help understanding and using perfective aspect with activity predicates and such learning may have a critical acquisition stage: The U-shaped developmental pattern observed in the study suggests that it may be especially effective to introduce aspect markers’ varied usages including non-prototypical associations to learners who are approaching the advanced level. Finally, as typical language use in the classroom may show biased patterns of predicate types, opportunities should be created whenever possible to immerse learners with naturalistic language input outside the classroom to achieve optimal integration of in-class and real-world language practices. For instance, Gong et al. (2021) reported that students’ formal learning in the classroom can be enhanced by strategic effort of practicing language outside the classroom in study abroad settings. Since our study shows that learner patterns are influenced by frequency biases, there is reason to believe that naturalistic exposure can help learners comprehend and use -*le* structures in more native-like ways.

## Conclusion

Investigating the effects of grammatical and lexical aspects on L2 acquisition and processing of temporality by L2 Chinese learners through a combination of online and offline comprehension tasks, we found that (1) imperfectives overall were processed faster across the board, explained by the relative semantic complexity of aspect markers and their syntactic properties; (2) grammatical and lexical aspect in general interactively constrain L2 aspect acquisition and processing, accounted for by semantic congruency and input frequency; (3) a prototypical grammatical aspect effect was evident for activities but less so for accomplishments in the L2 group across tasks, and (4) L2 proficiency and WMC were observed to modulate certain processes such as differences between the two grammatical aspects or lexical aspects. The study has several limitations. First, because the role of proficiency level in L2 performances examined was based on a rather small sample in each learner group, the interpretations regarding the influence of L2 proficiency are tentative. Future studies could also test a larger sample of L2 learners with a wider range of proficiency to fully investigate potential proficiency effects. Another issue that warrants further systematic research is the influence of WMC. WMC is a complex construct, and accurately measuring WMC is not an easy task (Juffs and Harrington, 2011). Given WMC’s multi-faceted nature, future studies could assess WMC using a battery of tests, and outcomes from each test could be aggregated for an overall estimate of WMC of the population in question (see Huettig and Janse, 2016 for an example). Finally, learners’ language exposure experience (heritage vs. non-heritage) was examined for its effect only as an exploratory analysis in the current study and is worth investigating more fully in future research.

## Data availability statement

The datasets presented in this study can be found in online repositories. The names of the repository/repositories and accession number(s) can be found at: OSF: https://osf.io/h2m5a/.

## Ethics statement

The studies involving human participants were reviewed and approved by IRB of University of Pittsburgh. The patients/participants provided their written informed consent to participate in this study.

## Author contributions

Both authors have made substantial, direct, and intellectual contributions to the work and approved it for publication.

## Funding

The open access publication fee for this article was fully paid by the University Library System, University of Pittsburgh, to which we would like to express our sincere thanks.

## Conflict of interest

The authors declare that the research was conducted in the absence of any commercial or financial relationships that could be construed as a potential conflict of interest.

## Publisher’s note

All claims expressed in this article are solely those of the authors and do not necessarily represent those of their affiliated organizations, or those of the publisher, the editors and the reviewers. Any product that may be evaluated in this article, or claim that may be made by its manufacturer, is not guaranteed or endorsed by the publisher.

## Footnotes

^1^"?" is used to indicate that the sentence may be marginally acceptable.

^2^The in-house OPIs refer to American Council on the Teaching of Foreign Languages (ACTFL) OPIs, see ACTFL Proficiency Guidelines (2012).

^3^The second author of the study is an experienced Chinese language instructor who is familiar with the participants’ instructional materials.

^4^CL is the abbreviation for classifier.

^5^Admittedly, it would be ideal for the sentence length to be counterbalanced across conditions. However, sentences containing ACCs, with the addition of two characters for the numeral and the classifier (e.g., *yi-kuai* ‘a piece of’ as in *zuo yi-kuai dangao* ‘make a piece of cake’), are inherently longer than those containing ACTs, as ACTs contain only the bare verb and the bare noun [e.g., *zuo dangao* ‘make cake(s)’]. This confound was properly handled in statistical modeling. See footnote 8.

^6^ https://gorilla.sc/

^7^We are very grateful to Foong Ha Yap for her kindness in sharing her materials with us.

^8^For both the sentence-picture matching task and the translation task, only the sentences for the L2 group were complemented with *pinyin*; Chinese native speakers read plain Chinese texts.

^9^ https://osf.io/h2m5a/

^10^The Bilingual Turn advocates that scholars in the field should reconsider some approaches taken to the study of SLA (Ortega, 2009). One proposed approach is that L2 learners should be investigated in their own right, not as deficient or deviant replicas of native speakers.

^11^Sentence length and its interaction with other fixed factors included in the models as the covariates revealed no significant effects for any model (all *p* > 0.05), hence excluding this potential confound.

^12^With such coding schemes, intercepts represent the grand mean rather than the mean for the baseline as determined otherwise by the default treatment contrasts in R. Treatment contrasts are not recommended for models where interactions are included, as it would lead to results being difficult to interpret (Singmann and Kellen, 2019).

## References

[ref1] ACTFL Proficiency Guidelines (2012). Alexandria, VA: American Council on the Teaching of Foreign Languages.

[ref2] Altmann G. T. Kamide Y. (2007). The real-time mediation of visual attention by language and world knowledge: linking anticipatory (and other) eye movements to linguistic processing. J. Mem. Lang. 57, 502–518. doi: 10.1016/j.jml.2006.12.004

[ref4] Andersen R. W. Shirai Y. (1994). Discourse motivations for some cognitive acquisition principles. Stud. Second. Lang. Acquis. 16, 133–156. doi: 10.1017/S0272263100012845

[ref5] Andersen R. W. Shirai Y. (1996). “The primacy of aspect in first and second language acquisition: the pidgin-creole connection,” in Handbook of Second Language Acquisition. eds. Ritchie W. C. Bhatia T. K. (San Diego, CA: Academic Press), 527–570.

[ref6] Anderson R. (1993). “Four operating principles and input distribution as explanations for underdeveloped and mature morphological systems,” in Progression and Regression in Language. eds. Hyltenstam K. Viborg A. (Cambridge: CUP), 309–339.

[ref7] Anwyl-Irvine A. L. Massonnié J. Flitton A. Kirkham N. Evershed J. K. (2020). Gorilla in our midst: an online behavioral experiment builder. Behav. Res. Methods 52, 388–407. doi: 10.3758/s13428-019-01237-x, PMID: 31016684 PMC7005094

[ref8] Baayen R. H. Davidson D. J. Bates D. M. (2008). Mixed-effects modeling with crossed random effects for subjects and items. J. Mem. Lang. 59, 390–412. doi: 10.1016/j.jml.2007.12.005

[ref9] Bardovi-Harlig K. (2000). Tense and aspect in second language acquisition: form, meaning, and use. Lang. Learn. 50, 11–13.

[ref10] Bardovi-Harlig K. (2012). “After process, then what? A longitudinal investigation of the progressive prototype in L2 English,” in Tense, aspect and mood in First and Second Language Acquisition. ed. Labeau E. (Leiden: Brill), 131–151.

[ref11] Bardovi-Harlig K. Comajoan-Colomé L. (2020). The aspect hypothesis and the acquisition of L2 past morphology in the last 20 years: a state-of-the-scholarship review. Stud. Second. Lang. Acquis. 42, 1137–1167. doi: 10.1017/S0272263120000194

[ref12] Bardovi-Harlig K. Reynolds D. W. (1995). The role of lexical aspect in the acquisition of tense and aspect. TESOL Q. 29, 107–131. doi: 10.2307/3587807

[ref13] Bassetti B. Lu M. (2016). Effects of interword spacing on native English readers of Chinese as a second language. Int. Rev. Appl. Linguist. Lang. Teach. 54, 1–22. doi: 10.1515/iral-2016-0014

[ref14] Bates D. Mächler M. Bolker B. Walker S. (2015). Fitting linear mixed-effects models using lme4. J. Stat. Softw. 67, 1–48. doi: 10.18637/jss.v067.i01

[ref15] Becker R. B. Ferretti T. R. Madden-Lombardi C. J. (2013). Grammatical aspect, lexical aspect, and event duration constrain the availability of events in narratives. Cognition 129, 212–220. doi: 10.1016/j.cognition.2013.06.014, PMID: 23942347

[ref16] Campbell S. (2014). Translation into the Second Language. London: Routledge.

[ref17] Chan H. L. (2012). *Tense-Aspect Processing in Second Language Learners*. Doctoral dissertation, University of Pittsburgh, Pittsburgh, PA.

[ref18] Chen J. Shirai Y. (2010). The development of aspectual marking in child mandarin Chinese. Appl. Psycholinguist. 31, 1–28. doi: 10.1017/S0142716409990257

[ref19] Cohen J. (1960). A coefficient of agreement for nominal scales. Educ. Psychol. Meas. 20, 37–46. doi: 10.1177/001316446002000104

[ref20] Collins L. (2002). The roles of L1 influence and lexical aspect in the acquisition of temporal morphology. Lang. Learn. 52, 43–94. doi: 10.1111/1467-9922.00177

[ref21] Comajoan L. (2006). The aspect hypothesis: development of morphology and appropriateness of use. Lang. Learn. 56, 201–268. doi: 10.1111/j.0023-8333.2006.00347.x

[ref22] Comrie B. (1976). *Aspect: An Introduction to the Study of Verbal Aspect and Related Problems*, *Vol. 2.* Cambridge: Cambridge University Press.

[ref23] Cook V. (2008). Multi-competence: black hole or wormhole for second language acquisition research. Understand. Sec. Lang. Proc. 25, 16–26.

[ref24] Core Team R. (2021). *R: A Language and Environment for Statistical Computing*. R Foundation for Statistical Computing.

[ref26] Dowty D. R. (1979). *Word Meaning and Montague Grammar. The Semantics of Verbs and Times in Generative Semantics and in Montague’s PTQ*. Dordrecht: Reidel. Studies in Linguistics and Philosophy, 7.

[ref27] Duff P. Li D. (2002). The acquisition and use of perfective aspect in Mandarin. Lang. Acquisit. Lang. Dis. 27, 417–454. doi: 10.1075/lald.27.17duf

[ref28] Fang S. Yuan F. (2021). Constraints of lexical and grammatical aspect on event representations in Mandarin Chinese. ExLing 2021, 77–80. doi: 10.36505/ExLing-2021/12/0020/000493

[ref30] Gabriele A. (2009). Transfer and transition in the SLA of aspect: a bidirectional study of learners of English and Japanese. Stud. Second. Lang. Acquis. 31, 371–402. doi: 10.1017/S0272263109090342

[ref31] Gao X. S. Liao Y. L. Li Y. X. (2014). Empirical studies on foreign language learning and teaching in China (2008–2011): a review of selected research. Lang. Teach. 47, 56–79. doi: 10.1017/S0261444813000414

[ref32] Godfroid A. Loewen S. Jung S. Park J. H. Gass S. Ellis R. (2015). Timed and untimed grammaticality judgments measure distinct types of knowledge: evidence from eye-movement patterns. Stud. Second. Lang. Acquis. 37, 269–297. doi: 10.1017/S0272263114000850

[ref33] Gong Y. Gao X. Lyu B. (2020a). Teaching Chinese as a second or foreign language to non-Chinese learners in mainland China (2014–2018). Lang. Teach. 53, 44–62. doi: 10.1017/S0261444819000387

[ref004] Gong Y. Guo Q. Li M. Lai C. Wang C. (2021). Developing literacy or focusing on interaction: New Zealand students’ strategic efforts related to Chinese language learning during study abroad in China. System 98:102462. doi: 10.1016/j.system.2021.102462

[ref34] Gong Y. Lai C. Gao X. (2020b). The teaching and learning of Chinese as a second or foreign language: The current situation and future directions. Front. Educ. China 15, 1–13. doi: 10.1007/s11516-020-0001-0

[ref001] Hendriks H. (1999). The acquisition of temporal reference in first and second language acquisition: what children already know and adults still have to learn and vice versa. Psychol. Lang. Commun. 3, 41–60.

[ref35] Huettig F. Janse E. (2016). Individual differences in working memory and processing speed predict anticipatory spoken language processing in the visual world. Lang. Cogn. Neurosci. 31, 80–93. doi: 10.1080/23273798.2015.1047459

[ref36] Jaeger T. F. (2008). Categorical data analysis: away from ANOVAs (transformation or not) and towards logit mixed models. J. Mem. Lang. 59, 434–446. doi: 10.1016/j.jml.2007.11.007, PMID: 19884961 PMC2613284

[ref37] Jin L. Hendriks H. (2005). The development of aspect marking in L1 and L2 Chinese. Work. Papers English Appl. Linguist. 9, 69–99.

[ref38] Jing-Schmidt Z. Lang J. Shi H. H. Hung S. H. Zhu L. (2022). Aspect construal in mandarin: a usage-based constructionist perspective on LE. Linguistics 60, 541–577. doi: 10.1515/ling-2020-0198

[ref39] Juffs A. Harrington M. (2011). Aspects of working memory in L2 learning. Lang. Teach. 44, 137–166. doi: 10.1017/S0261444810000509

[ref42] Labeau E. (2005). Beyond the aspect hypothesis: tense–aspect development in advanced L2 French. EUROSLA Yearb. 5, 77–101. doi: 10.1075/eurosla.5.06lab

[ref43] Lee E. J. (2001). Interlanguage development by two Korean speakers of English with a focus on temporality. Lang. Learn. 51, 591–633. doi: 10.1111/0023-8333.00169

[ref44] Lee E. Kim H. Y. (2007). On crosslinguistic variations in imperfective aspect: the case of L2 Korean. Lang. Learn. 57, 651–685. doi: 10.1111/j.1467-9922.2007.00431.x

[ref002] Lenth R. (2020). Emmeans: Estimated Marginal Means aka Least-Squares Means. R package Version 1.7.0. Available online at: https://cran.r-project.org/package=emmeans (Accessed May 2022).

[ref45] Li P. (1990). *Aspect and Aktionsart in Child Mandarin*. Unpublished doctoral dissertation, University of Leiden.

[ref46] Li H. Liang L. Wu D. (2022). Predicting Chinese preschoolers’ acquisition of aspect markers: a corpus-based study. Languages 7:133. doi: 10.3390/languages7020133

[ref47] Li P. Shirai Y. (2000). *The Acquisition of Lexical and Grammatical Aspect* (*Vol. 16*). Berlin: Walter de Gruyter.

[ref50] Linck J. A. Cunnings I. (2015). The utility and application of mixed-effects models in second language research. Lang. Learn. 65, 185–207. doi: 10.1111/lang.12117

[ref51] Linck J. A. Osthus P. Koeth J. T. Bunting M. F. (2014). Working memory and second language comprehension and production: a meta-analysis. Psychon. Bull. Rev. 21, 861–883. doi: 10.3758/s13423-013-0565-2, PMID: 24366687

[ref52] Liu F. H. (2012). L2 acquisition of the progressive marker zai in Mandarin Chinese. Chin. Sec. Lang. Res. 1, 153–192. doi: 10.1515/caslar-2012-0011

[ref54] Loewen S. (2009). “4. Grammaticality judgment tests and the measurement of implicit and explicit L2 knowledge,” in Implicit and Explicit Knowledge in Second Language Learning, Testing and Teaching. eds. Ellis R. Loewen S. Elder C. Erlam R. Philp J. Reinders H. (Tonawanda, NY: Multilingual Matters), 94–112.

[ref55] Lüdecke D. (2021). sjPlot: Data Visualization for Statistics in Social Science. R package version 2.8.9. Available at: https://CRAN.R-project.org/package=sjPlot

[ref56] Madden C. J. Zwaan R. A. (2003). How does verb aspect constrain event representations? Mem. Cogn. 31, 663–672. doi: 10.3758/BF03196106, PMID: 12956232

[ref57] Magliano J. P. Schleich M. C. (2000). Verb aspect and situation models. Discourse Process. 29, 83–112. doi: 10.1207/S15326950dp2902_1

[ref59] McManus K. (2013). Prototypical influence in second language acquisition: what now for the aspect hypothesis. Int. Rev. Appl. Linguist. Lang. Teach. 51, 299–322. doi: 10.1515/iral-2013-0013

[ref63] Montrul S. Slabakova R. (2003). Competence similarities between native and near-native speakers: an investigation of the preterite-imperfect contrast in Spanish. Stud. Second. Lang. Acquis. 25, 351–398. doi: 10.1017/S0272263103000159

[ref64] Orfitelli R. Polinsky M. (2017). When performance masquerades as comprehension: grammaticality judgments in experiments with non-native speakers. In *Quantitative approaches to the Russian language*. (London: Routledge), 197–214.

[ref65] Ortega L. (2009). Understanding Second Language Acquisition. London: Hodder Education.

[ref67] Rızaoğlu F. Gürel A. (2020). Second language processing of English past tense morphology: the role of working memory. Int. Rev. Appl. Linguist. Lang. Teaching. doi: 10.1515/iral-2019-0005

[ref68] Roberts L. (2012). Individual differences in second language sentence processing. Lang. Learn. 62, 172–188. doi: 10.1111/j.1467-9922.2012.00711.x

[ref69] Roberts L. Liszka S. A. (2013). Processing tense/aspect-agreement violations on-line in the second language: a self-paced reading study with French and German L2 learners of English. Second. Lang. Res. 29, 413–439. doi: 10.1177/0267658313503171

[ref71] Ryu J. Y. Horie K. Shirai Y. (2015). Acquisition of the Korean imperfective aspect markers –ko iss–and–a iss–by Japanese learners: a multiple-factor account. Lang. Learn. 65, 791–823. doi: 10.1111/lang.12132

[ref40] Ryu J. Shirai Y. (2022). L1 acquisition of the tense-aspect markers -ess (past-perfective) and -ko iss (imperfective) in Korean. J. Child Lang. 1–21. doi: 10.1017/S0305000922000113 35491939

[ref72] Salaberry M. R. (1999). The development of past tense verbal morphology in classroom L2 Spanish. Appl. Linguis. 20, 151–178. doi: 10.1093/applin/20.2.151

[ref73] Salaberry M. R. (2000). The acquisition of English past tense in an instructional setting. System 28, 135–152. doi: 10.1016/S0346-251X(99)00065-2

[ref003] Shirai Y. Andersen R. W. (1995). The acquisition of tense-aspect morphology: a prototype account. Language 71, 743–762. doi: 10.2307/415743

[ref76] Shirai Y. (1995). Tense-aspect marking by L2 learners of Japanese. In Proceedings of the 19th Annual Boston University Conference on Language Development (*Vol. 2*), pp. 575–586). Massachusetts, US: Cascadilla Press.

[ref77] Shirai Y. (1998). The emergence of tense-aspect morphology in Japanese: universal predisposition? First Lang. 18, 281–309. doi: 10.1177/014272379801805403

[ref79] Shirai Y. (2002). The prototype hypothesis of tense-aspect acquisition in second language. Lang. Acquisit. Lang. Disord. 27, 455–478. doi: 10.1075/lald.27.18shi

[ref80] Shirai Y. (2004). A multiple-factor account for the form-meaning connections in the acquisition of tense-aspect morphology. *Form-meaning connections in second language acquisition*. (Hillsdale, NJ: Lawrence Erlbaum), 91–112.

[ref81] Shirai Y. (2010). Semantic bias and morphological regularity in the acquisition of tense-aspect morphology: what is the relation? Linguistics 48, 171–194. doi: 10.1515/ling.2010.005

[ref82] Shirai Y. Kurono A. (1998). The acquisition of tense-aspect marking in Japanese as a second language. Lang. Learn. 48, 279–244. doi: 10.1111/1467-9922.00041

[ref83] Singmann H. Kellen D. (2019). An introduction to mixed models for experimental psychology. New Methods Cogn. Psychol. 28, 4–31. doi: 10.4324/9780429318405-2

[ref84] Smith C. S. (1997). The Parameter of Aspect (2nd Edn.). Dordrecht: Kluwer.

[ref85] Stoll S. (1998). The role of Aktionsart in the acquisition of Russian aspect. First Lang. 18, 351–376. doi: 10.1177/014272379801805405

[ref86] Sugaya N. Shirai Y. (2007). The acquisition of progressive and resultative meanings of the imperfective aspect marker by L2 learners of Japanese: transfer, universals, or multiple factors? Stud. Second. Lang. Acquis. 29, 1–38. doi: 10.1017/S0272263107070015

[ref89] Tong X. Shirai Y. (2016). L2 acquisition of mandarin zai and –le. Chin. Sec. Lang. Res. 5, 1–25. doi: 10.1515/caslar-2016-0001

[ref90] Van Hout A. (2008). Acquiring perfectivity and telicity in Dutch, Italian and Polish. Lingua 118, 1740–1765. doi: 10.1016/j.lingua.2007.08.011

[ref92] Vendler Z. (1967). Linguistics in Philosophy. Ithaca, NY: Cornell University Press

[ref93] Wechsler D. (1981). Wechsler Adult Intelligence Scale, Revised Manual. New York, NY: Psychological Corp.

[ref94] Wen X. (1995). Second language acquisition of the Chinese particle le. Int. J. Appl. Linguist. 5, 45–62. doi: 10.1111/j.1473-4192.1995.tb00072.x

[ref95] Wen X. (1997). Acquisition of Chinese aspect: AN analysis of the interlanguage of learners of Chinese as a foreign language. ITL Int. J. Appl. Linguist. 117–118, 1–26. doi: 10.1075/itl.117-118.01wen

[ref96] Wen Z. (2015). Working memory in second language acquisition and processing: The phonological/executive model. *Working memory in second language acquisition and processing*. (Bristol: Multilingual Matters), 41–62.

[ref97] Wickham H. Averick M. Bryan J. Chang W. McGowan L. François R.. (2019). Welcome to the tidyverse. J. Open Sour. Soft. 4:1686. doi: 10.21105/joss.01686

[ref98] Xiao R. McEnery T. (2004). Aspect in Mandarin Chinese: a corpus-based study (Vol. 73) Amsterdam: John Benjamins Publishing.

[ref99] Xu Y. (2020). Perfective-le use and consciousness-raising among beginner-level Chinese learners. Languages 5:16. doi: 10.3390/languages5020016

[ref100] Yang S. (2016). “Aspect hypothesis” and L2 acquisition of aspect markers-le and -zhe. Chin. Teach. World 30, 101–118.

[ref102] Yap F. H. Chu P. C. K. Yiu E. S. M. Wong S. F. Kwan S. W. M. Matthews S.. (2009). Aspectual asymmetries in the mental representation of events: role of lexical and grammatical aspect. Mem. Cogn. 37, 587–595. doi: 10.3758/MC.37.5.587, PMID: 19487750

[ref103] Zeng X. Chen X. Shirai Y. (2021). Lexical and grammatical aspect in on-line processing of English past tense and progressive aspect by mandarin speakers. Front. Psychol. 12:661923. doi: 10.3389/fpsyg.2021.661923 34177715 PMC8222903

